# Paradoxical effects of low dose MDMA on latent inhibition in the rat

**DOI:** 10.1016/j.neuropharm.2012.11.012

**Published:** 2013-04

**Authors:** A.J.D. Nelson, K.E. Thur, C.A. Marsden, H.J. Cassaday

**Affiliations:** a School of Psychology, University of Nottingham, University Park, Nottingham, NG7 2RD, United Kingdom; b School of Biomedical Sciences, University of Nottingham, United Kingdom

**Keywords:** MDMA, 5-Hydroxytyptamine, Latent inhibition, HPLC-ED

## Abstract

The cognitive effects of MDMA (‘Ecstasy’) are controversial, particularly in the case of acute administration of low doses. Latent inhibition (LI) refers to the reduction in conditioning to a stimulus that has received non-reinforced pre-exposure, an effect typically abolished by amphetamines and enhanced by antipsychotics. LI enhancement has also been shown using the 5-HT reuptake blocker sertraline. In the present study, the effects of MDMA (6 mg/kg, known to increase 5-HT release) were tested using 10 and 40 pre-exposures to produce weak and strong LI in controls, respectively. MDMA (injected twice, prior to pre-exposure and conditioning) significantly enhanced LI in that the effect was clearly demonstrated after only 10 pre-exposures, when it was absent in the saline controls. On its own such a profile of action would be consistent with a procognitive effect of MDMA mediated by increased availability of 5-HT. However, paradoxically the same MDMA treatment reduced LI in the 40 pre-exposures condition. This component of action is likely attributable to MDMA's actions on catecholaminergic systems and is consistent with other evidence of its adverse effects. Moreover, there were small but significant reductions in 5-HT in medial prefrontal cortex (mPFC) and amygdala assayed 7 days post MDMA administration (2 × 6 mg/kg, 24 h apart).

## Highlights

► MDMA (6 mg/kg) enhances latent inhibition produced by 10 stimulus pre-exposures. ► MDMA (6 mg/kg) abolishes latent inhibition produced by 40 stimulus pre-exposures. ► HPLC analyses reveal evidence of 5-HT depletion after 2 × 6 mg/kg doses of MDMA.

## 1 Introduction

Latent inhibition (LI) normally manifests as poorer conditioning to a stimulus that has been previously presented without consequence (Lubow and Moore, 1959). This effect is abolished by treatment with the indirect catecholamine agonist amphetamine (e.g., Solomon et al., 1981; Weiner et al., 1981, 1984, 1988; Gray et al., 1992; Kumari et al., 1999; Nelson et al., 2011a). LI is similarly abolished by electrolytic lesions to the medial raphe, source of the ascending projections of the indoleamine 5-hydroxytryptamine (serotonin, 5-HT) (Solomon et al., 1980), as well as by selective 5-HT depletion using the neurotoxin 5,7-dihydroxytyptamine (5,7-DHT) (Loskutova et al., 1990; Cassaday et al., 1993a; Nelson et al., 2012), and by pharmacological treatments which reduce 5-HT release (Solomon et al., 1978) or block its actions at postsynaptic 5-HT2 receptors (Cassaday et al., 1993b; Shadach et al., 2000). Conversely, LI enhancement – demonstrated when LI in controls has been systematically weakened using a reduced number of stimulus pre-exposures – has been shown using the 5-HT reuptake blocker (selective serotonergic reuptake inhibitor, SSRI) sertraline (Loskutova, 1998). LI is similarly enhanced by dopaminergic antipsychotics (Weiner and Feldon, 1987; Feldon and Weiner, 1991; Dunn et al., 1993), thus that the equivalent effect can be produced by treatment with an SSRI is consistent with the procognitive actions of SSRIs.

However, the picture is complex in that LI enhancement has also been reported after treatment with 5-HT2 antagonists (McDonald et al., 2003) as well as by deafferentation of the 5-HT input to the shell subregion of the nucleus accumbens (NAc) (Nelson et al., 2012) whereas the 5-HT2 agonist DOI has been shown to disrupt LI (Hitchcock et al., 1997). To our knowledge, further positive effects of treatment with pharmacological agents which mimic the actions of 5-HT postsynaptically have yet to be demonstrated.

3,4-Methylenedioxymethamphetamine (MDMA, ‘Ecstasy’) is a distinctive member of the amphetamine family, both in terms of its neurochemical profile and associated psychological effects. In particular, the increased release of 5-HT produced by the controlled administration of MDMA in therapeutic settings has been reported to produce an anxiolytic effect and other pleasurable consequences related to its designation as an entactogen (Bouso et al., 2008; Johansen and Krebs, 2009). However, in animal studies, the repeated use of MDMA (or the administration of higher doses, Green et al., 2009) has been shown to lead to a range of acute adverse effects from hyperthermia to death (Green et al., 2003). In addition, using established neuropsychological test batteries, cognitive deficits have been demonstrated in regular recreational Ecstasy users, although these are typically confounded by the purity of the currently available supply, polydrug use and pre-existing psychiatric problems (Fox et al., 2001; Soar et al., 2006). Again the influence of polydrug use is difficult to discount, but psychotic episodes have also been reported in cases of long-term recreational Esctasy consumption (McGuire and Fahy, 1991; Marchesi et al., 2009).

Relatively high doses of MDMA (10 and 15 mg/kg, repeated administrations) have recently been reported to be without effect on LI in a rat model (Skelton et al., 2012), but this was study of neurodevelopmental sensitivity which also used behavioural procedures different from those routinely used to examine the effects of amphetamine on LI (Weiner et al., 1981, 1984, 1988; Nelson et al., 2011a). The present study used a selected lower dose of MDMA (6 mg/kg i.p.) – previously demonstrated to increase extracellular 5-HT measured in rat hippocampus by microdialysis (Rodsiri et al., 2011) – and therefore suitable to further examine the role of 5-HT in LI. Moreover systemic administration of 6 mg/kg in the rat is within the range comparable to human doses (Sessa and Nutt, 2007; Green et al., 2009). The effects of this MDMA treatment were tested using a conditioned emotional response (CER) procedure, typical of those used in LI studies, and demonstrated to be sensitive to the effects of d-amphetamine, which as expected abolished LI (Nelson et al., 2011a). However, the present study also included a procedural variant (low number of stimulus pre-exposures) demonstrated suitable to show LI enhancement (Nelson et al., 2010, 2011b). Specifically, LI after 10 and 40 pre-exposures was used to produce respectively weak and strong LI in controls, and to examine the effects of MDMA thereon.

Because of the possibility that even low doses and acute administration of MDMA can result in adverse effects which have been argued to be related to neurotoxicity (Fox et al., 2001; Green et al., 2003, 2009; Soar et al., 2006) tissue samples were taken for neurochemical assay as soon as possible upon completion of the behavioural tests.

## 2 Materials and methods

### 2.1 Subjects

72 experimentally naïve adult male Wistar rats (Charles River, UK) were caged in pairs on a 12:12 h light/dark cycle with food and water *ad libitum*. They were handled for approximately 10 min per day for 1 week and then at mean weight 221 g (range 202–250 g) were placed on water deprivation immediately prior to behavioural procedures.

All procedures were carried out in accordance with the United Kingdom (UK) Animals Scientific Procedures Act 1986, Project Licence number: PPL 40/3163.

### 2.2 Drug administration

MDMA HCl (Tocris, UK) was dissolved in saline at 6 mg/ml (weight calculated as the free base) for injection (i.p.) at 1 ml/kg to administer a dose of 6 mg/kg. Control rats were injected with the equivalent volume of saline. Drug or control injections were administered 40 min prior to the pre-exposure and conditioning stages of the procedure (Joseph et al., 2000). Thus all rats given MDMA received 2 doses of 6 mg/kg with a 24 h gap between the two doses.

### 2.3 Behavioural apparatus

Six identical fully automated conditioning boxes, housed within sound-attenuating cases containing ventilation fans (Cambridge Cognition, Cambridge, UK), were used. The inner conditioning box walls consisted of plain steel (25 cm × 25 cm × 22 cm high) with a Plexiglas door (27 cm × 21 cm high), at the front. The floor was a shock grid with steel bars 1 cm apart and 1 cm above the lip of a 7 cm deep sawdust tray. A waterspout was mounted on one wall. The spout was 5 cm above the floor and connected to a lickometer supplied by a pump. Licks were registered by a break in the photo beam within the spout, which also triggered water delivery of 0.05 ml per lick. The waterspout was illuminated when water was available. A loudspeaker for the presentation of auditory stimuli was set in the roof. A 5 s mixed frequency noise set at 85 dB (including background) served as the CS. Footshock of 1 s duration and 1 mA intensity provided the unconditioned stimulus (UCS). This was delivered through the grid floor by a constant current shock generator (pulsed voltage: output square wave 10 ms on, 80 ms off, 370 V peak under no load conditions, MISAC Systems, Newbury, UK). Stimulus control and data collection was by an Acorn Archimedes RISC computer programmed in Basic with additional interfacing using an Arachnid extension (Cambridge Cognition).

### 2.4 Behavioural procedure

Water deprivation was introduced 1 day prior to shaping. Thereafter, the animals received 1hr and 15 min of *ad libitum* access to water in their home cage in addition to water in the experimental boxes. The stages of the CER procedure were as follows:

#### 2.4.1 Pre-training

In order to initiate licking behaviour, rats were placed in the conditioning boxes with their respective cage mate and were shaped for 1 day until all drank from the waterspout. No data were recorded. Thereafter, animals were individually assigned to a conditioning box for the duration of the experiment (counterbalanced by experimental group).

There then followed 5 days of pre-training, in which rats drank in their conditioning boxes for 15 min each day (timed from first lick). The drinking spout was illuminated throughout, but no other stimuli were presented in this phase. Latency to first lick was recorded to assess any pre-existing differences in readiness to drink (prior to conditioning).

#### 2.4.2 Pre-exposure

Animals were placed in their allocated boxes, where the pre-exposed animals received 10 5 s CS presentations with an average inter-stimulus interval of 60 s (PE10) or 40 5 s CS presentations with an average inter-stimulus interval of 60 s (PE40). In order to match box exposure between the groups, the 10PE group remained in the apparatus for a further 30 min without receiving any CS presentations. The non-pre-exposed control animals were confined to the apparatus for an identical period of time (40 min) without receiving any CS presentations. Water was not available within the box and the waterspout was not illuminated during the pre-exposure session.

#### 2.4.3 Conditioning

Conditioning was conducted on the day following pre-exposure. No water was available within the box and the waterspout was not illuminated. There were 2 conditioning trials in which the UCS footshock was delivered following termination of the CS. The first pairing of CS and UCS was presented after 5 min had elapsed, and the second pairing was 5 min after the first, followed by a further 5 min left in the apparatus. In the absence of drinking, there were no behavioural measures to record.

#### 2.4.4 Reshaping

On the day following conditioning, animals were reshaped following the same procedure as in pre-training sessions. This was in order to re-establish drinking after conditioning.

#### 2.4.5 Test

On the day following reshaping, the animals were placed in the conditioning boxes and underwent an extinction test to the CS. Water was available throughout the test and the waterspout was illuminated. Once the animals had made 50 licks, the CS was presented for 15 min. The latency to make 50 licks in the absence of the CS (the A period, timed from the first lick made in each box) provided a measure of any individual variation in baseline lick responding. This was compared with the time taken to complete 50 licks following CS onset (B period) in a suppression ratio (A/(A + B)) to assess the level of conditioning to the CS, adjusted for any individual variation in drink rate.

### 2.5 Design and analysis

There were 6 experimental groups run in a 3 × 2 independent factorial design with pre-exposure, at levels non-pre-exposed (NPE), 10 pre-exposures (10PE) and 40 pre-exposures (40PE), and drug, at levels saline and 6 mg/kg MDMA. Statistical analysis was performed using analysis of variance (ANOVA) with alpha set at *p* < 0.05 for the rejection of the null hypothesis. ANOVA of the pre-training latencies included the additional factor of days (at 5 levels). *T*-tests were used to explore significant interactions. The dependent variables were lick latencies at pre-training and reshaping, and the A period and suppression ratio for the test of conditioning. Where necessary, raw latency data (time to first lick at pre-training and reshape) were log transformed so that their distribution was suitable for parametric analysis.

### 2.6 Neurochemical assay

**Fig. 1:**
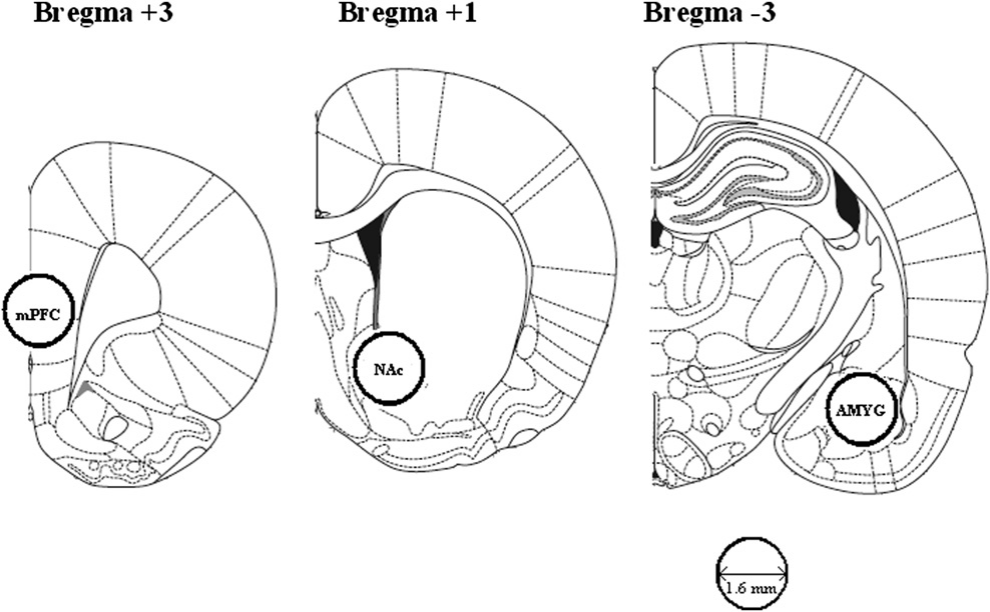
Forebrain regions dissected for postmortem neurochemical analysis. Regions of interest were dissected by pushing micropunch needles of 1.6 mm diameter into the posterior face of the coronal slices as indicated. Drawings are adapted from Paxinos and Watson (2005); numbers indicate distance from bregma in mm.

Tissue samples were taken, adapted from an established micro-punch procedure (Peleg-Raibstein et al., 2004; Nelson et al., 2010, 2011a, 2011b, 2012). Fig. 1 shows the location of the punches. One week after the first of the two MDMA injections, the behavioural control (NPE) rats (both saline- and MDMA-injected) were humanely killed by dislocation of the neck and decapitated. The brains were removed rapidly and were placed dorsal side up in an ice-chilled rat brain matrix (Harvard Instruments, USA).

Using ice-chilled razor blades, three 2 mm coronal brain sections were cut. The posterior side of the slices corresponded to approximately +3, +1 and −3 mm from bregma according to the atlas of Paxinos and Watson (2005). The brain samples were then immediately frozen on dry ice and stored at −80 °C. Subsequently, the three 2 mm coronal sections were placed posterior side up onto an ice-chilled plate. From the first section (+3 mm) a 1.6 mm diameter stainless steel micropunch was used to remove the medial prefrontal cortex (mPFC). From the second section (+1 mm), the 1.6 mm diameter stainless steel micropunch was also used to remove the NAc. From the third section (−3 mm) a 1.6 mm diameter stainless steel micropunch was used to remove the amygdala. For the NAc and amygdala samples, one punch was used per brain hemisphere, but the mPFC punch was taken across both hemispheres. Tissue punch samples were stored in sealed 1.5 ml Eppendorf tubes and frozen at −80 °C.

Neurotransmitter and metabolite levels in the samples were determined by high-pressure liquid chromatography with electrochemical detection (HPLC-ED). The tissue samples were homogenised in 0.1 M PCA solution by sonication and centrifuged at 17,400 g for 20 min at 4 °C before amine and metabolite levels were detected using a glassy carbon electrode flow cell (VT-03 Antec) with an ISAAC reference electrode. An external standard consisting of 5-HT, DA, and metabolites, in concentrations of 10^−7^, 0.5×10^−7^ and 10^−8^ M was injected at a volume of 4 μl for calibration. Samples were injected onto the column at 4 μl volumes.

Results were analysed using Alexys software data system. Bradford assay was used to adjust for protein content using the pellet remaining after sample centrifugation, adapted from previous methods (Bradford, 1976). The significance of changes in neurotransmitter levels and their metabolites was determined by *t*-test.

## 3 Results

### 3.1 Behavioural

#### 3.1.1 Pre-training

As would be expected, latencies to drink decreased over the duration of pre-training, reflected in a main effect of days, *F*_(4,264)_ = 18.36, *p* < 0.001. However there was no indication of any difference in readiness to drink in the conditioning boxes as a function of either pre-exposure or drug condition-to-be, either overall or in interaction with days, maximum *F*_(2,66)_ = 1.55 *p* = 0.22.

#### 3.1.2 Reshaping

**Fig. 2:**
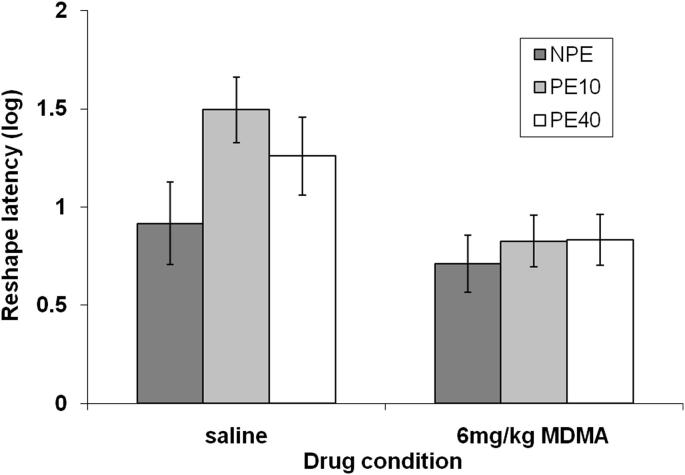
Mean latency (log s) to drink in the experimental context at the reshape session (±S.E.M.) for previously non-pre-exposed (NPE) rats (dark grey bars) and rats given 10 (PE10; light grey bars) or 40 pre-exposures (PE40; white bars).

There was no effect of pre-exposure or pre-exposure by drug interaction on latency to drink at reshape, maximum *F*_(2,66)_ = 2.25 *p* = 0.11. However, there was a main effect of drug, *F*_(1,66)_ = 10.19, *p* = 0.002, reflecting overall shorter latencies to drink in rats previously treated with MDMA (Fig. 2). This increased readiness to drink is consistent with reduced contextual fear conditioning.

#### 3.1.3 Test

**Fig. 3:**
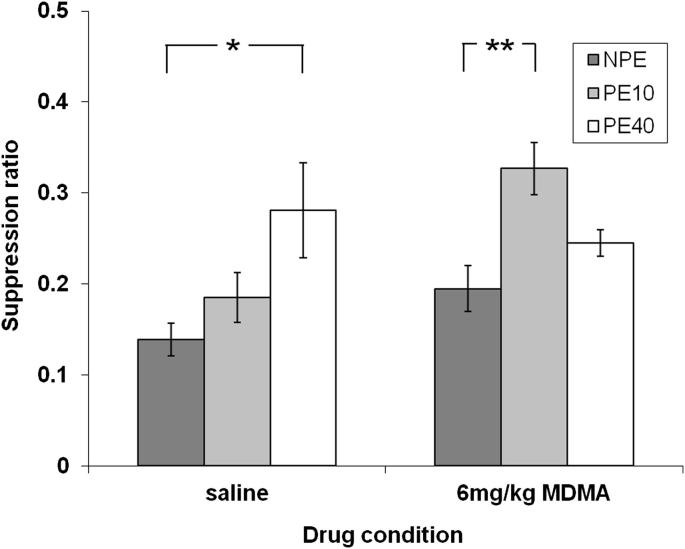
Mean suppression ratio (±S.E.M.) to the CS for previously non-pre-exposed (NPE) rats (dark grey bars) and rats given 10 (PE10; light grey bars) or 40 pre-exposures (PE40; white bars). Comparisons show significant latent inhibition; *p < 0.05; **p < 0.001 by *t*-test.

Prior to presentation of the CS, rats took overall comparable times to make 50 licks during the A period, by both drug and pre-exposure condition, maximum *F*_(2,66)_ = 1.19 *p* = 0.31. On the suppression ratio measure of learning, which takes individual variation in A period responding into account, there was a main effect of pre-exposure, *F*_(2,66)_ = 6.29, *p* = 0.003, reflecting an overall reduction in learning in consequence of prior non-reinforced experience with the CS. However, the previous MDMA treatments clearly affected the level of learning as there was a main effect of drug, *F*_(1,66)_ = 4.79, *p* < 0.05, as MDMA-treated rats were overall less suppressed than vehicle-injected controls. There was also a drug by pre-exposure interaction, *F*_(2,66)_ = 4.39, *p* < 0.05, indicating that the level of suppression to the CS differed by drug and conditioning group. Fig. 3 shows that (as expected) whilst LI was robust after 40 pre-exposures in the saline-injected controls, *t*_(22)_ = 2.57, *p* = 0.02, there was no evidence of LI after 10 pre-exposures in the saline-injected controls, *t*_(22)_ = 1.39, *p* = 0.18. By contrast, MDMA appeared to potentiate LI under conditions of weak pre-exposure as there was markedly less suppression to the CS in the PE10 relative to the NPE group, *t*_(22)_ = 3.46, *p* = 0.002. Conversely, with experimental parameters (40 pre-exposures) that produced robust LI in vehicle-injected animals, MDMA reduced LI as there was no statistical evidence that these animals conditioned less to the CS compared to the MDMA NPE group (*t*_(22)_ = 1.73, *p* = 0.97).

### 3.2 Effects of MDMA administration on amine and metabolite levels

The results of the neurochemical analysis by HPLC revealed that one vehicle-injected animal had abnormally high levels of DA in the mPFC (4.5 SD beyond the mean) and consequently its mPFC DA data were not included in the subsequent statistical analysis. Table 1 shows the absolute levels (pmoles/μg protein) of 5-HT, DA, 5HIAA and DOPAC in the 3 brain regions (mPFC, NAc and amygdala) from which samples were taken 7 days after drug. Table 2 shows the percentage depletion relative to the vehicle-injected control levels. There were small but significant reductions in 5-HT levels in the mPFC (*t*_(22)_ = 2.74, *p* < 0.05) and amygdala (*t*_(22)_ = 2.37, *p* < 0.01).

## 4 Discussion

The current experiment investigated the effects of acute treatment with MDMA on LI, which was tested using two sets of experimental parameters (PE10 and PE40) designed to preclude and secure the emergence of LI in vehicle-injected controls, respectively. Consistent with previous reports, in the PE40 LI group there was significantly less learning compared to the NPE control group, in other words, there was a robust LI effect in vehicle-injected controls. In the PE10 group, vehicle-injected rats did not show LI and conditioned to the PE stimulus at equivalent levels to the NPE stimulus. However, after acute treatment with MDMA (6 mg/kg i.p.) significant LI was demonstrated in the PE10 group. In other words, MDMA enhanced LI using experimental parameters designed to prevent the emergence of LI in vehicle-controls (PE10).

Previously, both systemic treatments which antagonise or deplete 5-HT (Solomon et al., 1978; Cassaday et al., 1993b; Shadach et al., 2000) and regionally specific 5-HT depletion have been shown to disrupt LI (Solomon et al., 1980; Loskutova et al., 1990; Loskutova, 2001; Cassaday et al., 1993a; Nelson et al., 2012). Thus, the demonstration here that acute treatment with MDMA enhances LI is entirely consistent with a modulatory role of 5-HT in the expression of LI and suggests that increased extracellular 5-HT function potentiates LI. This suggestion is supported by the earlier demonstration of increased LI after treatment with the SSRI sertraline (Loskutova, 1998).

An effect of MDMA was also identified at the reshaping stage of procedure. This took the form of an overall increased readiness to recommence drinking in the experimental context after the footshock conditioning on the previous day. Reduced contextual conditioning has been widely reported after treatment with SSRIs in other fear conditioning procedures (Inoue et al., 2011). Thus this component of MDMA action may reflect increased 5-HT release.

Conversely, LI was not demonstrated after MDMA treatment in the PE40 condition. At face value this seems a surprising result, but other compounds can produce opposing effects on LI depending on the experimental parameters used (Cassaday et al., 1993b; Hitchcock et al., 1997; Shadach et al., 2000; McDonald et al., 2003; Weiner, 2003; Barak and Weiner, 2007, 2009). Such opposing actions may also result in apparently null effects, and may explain why a recent study which included examination of the neurodevelopmental effects of MDMA reported no effects on LI, whereas other cognitive effects were clearly demonstrated (Skelton et al., 2012). However, there were also a number of important differences in the way LI and MDMA effects were assessed by Skelton et al. (2012), principally that animals were not conditioned under drug. Additionally, LI was assessed in a freezing procedure without any manipulation of the level of pre-exposure and after a pre-treatment regime of higher (10 mg/kg) and repeated doses of MDMA given at different developmental stages (Skelton et al., 2012).

As an amphetamine, MDMA is known to promote the release of DA (e.g. White et al., 1996) and NA (Rothman et al., 2001) as well as 5-HT, and treatment with indirect DA agonists is predicted to disrupt LI (Solomon et al., 1981; Weiner et al., 1981; Weiner et al., 1984, 1988; Gray et al., 1992; Kumari et al., 1999). Thus the pattern of results obtained in the present study is likely to be attributable to the acute effects of MDMA actions on both 5-HT and catecholamine systems. Further work will be necessary to draw firm conclusions as to which component of MDMA action mediates enhanced versus abolished LI. First, if mediated by increased DA release, the MDMA-induced abolition of strong LI should be reversible by treatment with typical antipsychotics such as haloperidol (Weiner and Feldon, 1987; Weiner, 2003; Weiner and Arad, 2009). If mediated by increased 5-HT release, the MDMA-induced enhancement of weak LI should be reversible by treatment with 5-HT antagonists. Second, the present study manipulated the level of LI using different numbers of stimulus pre-exposures (PE10 and PE40). An alternative method to manipulate the level of LI is to increase the number of conditioning trials to overcome the effect of pre-exposure and thus produce weak LI. The DA but not the 5-HT component of MDMA action would be predicted to be susceptible to this manipulation (Weiner, 2003; Weiner and Arad, 2009). Thus, in contrast to the clear effect seen in the PE10 group, MDMA should not enhance LI which has been weakened by increasing the number of conditioning trials.

Irrespective of the underlying mechanisms, on its own the demonstration of enhanced LI would be consistent with a procognitive effect of MDMA mediated by increased availability of 5-HT. However, the observation that the same MDMA treatment reduced LI in the 40 pre-exposures condition is consistent with other evidence of its adverse effects. Moreover, we found small but significant reductions in 5-HT upon completion of the present study. The MDMA administration regime was selected on the basis of its known acute effect as an indirect 5-HT agonist but its secondary effects would be expected to be different (Reneman et al., 2002). Previously, at 6 mg/kg MDMA, there was no evidence for loss of 5-HT function (measured 2–3 weeks post-administration) after 3 administrations over the course of a single day (Rodsiri et al., 2011). In the present study the cumulative dose of 12 mg/kg (2 × 6 mg/kg, 24 h apart) produced a small but significant reduction in 5-HT levels in the mPFC and amygdala, detected 1 week after the second MDMA administration. In addition to the interval to assay, the regions sampled were also different – we selected three relatively discrete regions on the basis of their inter-connectivity with hippocampus, as well as known involvement in LI (Weiner, 2003; Weiner and Arad, 2009; Nelson et al., 2010) – as were the strains of rats. In the Wistar strain, used in the present study, others have reported significant changes in the serotonergic system 1 week post administration though after a higher dose of 30 mg/kg MDMA (and in this case 5-HT_1A_ receptor density was shown to be increased by up to 30%; Aguirre et al., 1998). On a conservative estimate, the 6 mg/kg dose used in the present study would be equivalent to a dose of 2 mg/kg in humans (Green et al., 2009), thus very comparable to that used therapeutically without psychological or physiological adverse effects (Mithoefer, 2006; Bouso et al., 2008). However, the significant reduction in 5-HT that we found 7 days subsequent to a total dose of 12 mg/kg could be a contraindication. This finding – seen with a relatively low dose given acutely – suggests that evidence for the psychotherapeutic potential of MDMA should be treated with caution (Bouso et al., 2008; Johansen and Krebs, 2009; Parrott, 2007; Sessa and Nutt, 2007).

## Figures and Tables

**Table 1:** Mean absolute levels (±S.E.M) of 5-hydroxytryptamine (5-HT), 5-hydroxyindoleacetic acid (5-HIAA), dopamine (DA) and 3,4-dihydroxyphenylacetic acid (DOPAC) measured as (pmoles/μg protein). All samples were taken from behavioural control (non-pre-exposed) rats twice injected one week earlier with either saline or MDMA (6 mg/kg free base, i.p.) in the medial prefrontal cortex (mPFC), nucleus accumbens (NAc) and amygdala.

	5-HT		5-HIAA		DA		DOPAC	
	Saline	MDMA	Saline	MDMA	Saline	MDMA	Saline	MDMA
mPFC	0.192 (±0.018)	0.132 (±0.013)	0.157 (±0.022)	0.115 (±0.017)	0.045 (±0.004)	0.060 (±0.015)	0.016 (±0.003)	0.019 (±0.005)
NAc	0.416 (±0.038)	0.327 (±0.031)	0.470 (±0.017)	0.429 (±0.030)	5.727 (±0.400)	6.099 (±0.465)	2.088 (±0.162)	2.160 (±0.190)
Amygdala	0.531 (±0.039)	0.424 (±0.022)	0.404 (±0.026)	0.382 (±0.018)	1.305 (±0.237)	1.618 (±0.191)	0.249 (±0.046)	0.328 (±0.032)

**Table 2:** Mean percentage change (±S.E.M) in 5-hydroxytryptamine (5-HT), 5-hydroxyindoleacetic acid (5-HIAA), dopamine (DA) and 3,4-dihydroxyphenylacetic acid (DOPAC). All samples were taken from behavioural control (non-pre-exposed) rats twice injected one week earlier with either saline or MDMA (6 mg/kg free base, i.p.) in the medial prefrontal cortex (mPFC), nucleus accumbens (NAc) and amygdala. *Significant difference, *p* < 0.05, *t*-test.

	5-HT	5-HIAA	DA	DOPAC
mPFC	−31.2%* (±6.5)	−26.8% (±11.0)	+32.5% (±33.5)	+16.3% (±30.6)
NAc	−21.4% (±7.4)	−8.7% (±6.4)	+4.9% (±8.1)	+3.4% (±9.1)
Amygdala	−20.2%* (±4.2)	−5.4% (±4.4)	+24.0% (±14.6)	+31.9% (±12.8)
